# Stereoscopic plant-protection system integrating UAVs and autonomous ground sprayers for orchards

**DOI:** 10.3389/fpls.2022.1040808

**Published:** 2022-10-31

**Authors:** Shijie Jiang, Bingtai Chen, Wenwei Li, Shenghui Yang, Yongjun Zheng, Xingxing Liu

**Affiliations:** ^1^ College of Engineering, China Agricultural University, Beijing, China; ^2^ Yan Tai Institute, China Agricultural University, Yan Tai, China

**Keywords:** orchard, stereoscopic plant-protection, uniform spraying, UAV, UGV, computational fluid dynamics (CFD)

## Abstract

For orchard plant protection, conventional large machines and small sprayers are practically restricted by either narrow planting intervals with dense leaves or their inadequate penetration power, which leads to an unsatisfactory effect of spray. This paper proposes a stereoscopic plant-protection strategy that integrates unmanned air and ground sprayers to spray different parts of canopies to improve uniformity. In order to verify the proposal, a stereoscopic plant-protection system (SPS) was developed, consisting of a small swing-arm sprayer and a T16 plant-protection Unmanned Aerial Vehicle (UAV). Then, optimal operation parameters were determined by Computational Fluid Dynamics (CFD) and orthogonal experiments, and the uniformity was finally quantified by trials. CFD and orthogonal experiments showed that a swing-arm angle of 60° and a forward speed of 0.4 m/s were optimal for the ground sprayer, whilst a height of 2.0 m from the top of canopies and a forward speed of 1.0 m/s were appropriate for the UAV. The trial results showed that the density of vertical droplet deposition varied from 90 to 107 deposits/cm^2^ in canopies, and the uniformity was 38.3% higher than conventional approaches. The uniformity of top, bottom, inside and outside canopies was significantly improved. Meanwhile, the density of droplet deposition on both sides of leaves in all test points exceeded 25 deposits/cm^2^, able to meet the standard of spray. This study provides a practical approach for uniform pesticide spray to large-canopy fruit trees. Moreover, the high flexibility of plant-protection UAVs and the significant trafficability of small swing-arm sprayers can solve the problem of large machine entering and leaving orchards.

## 1 Introduction

Plant protection is important in the orchard production process to promote fruit production (Zhao et al., 2017; Jiang et al., 2021), while the current means still relies on chemical spray (Zhai et al., 2018; Rehberg et al., 2020; Zheng et al., 2020a). It is ideal for the entire canopy of fruit trees to be uniformly covered by droplets. However, due to topographical characteristics (such as undulating terrain, slope and unevenness) and narrow row intervals (especially row interval closure by canopies during tree branching and foliage densifying), large plant-protection machinery cannot enter orchards, while small one cannot achieve full-canopy spray. Thus, it faces serious difficulties for orchard plant protection to achieve expected effect (especially in hilly mountainous orchards) (Hołownicki et al., 2017; Zheng et al., 2020b).

Manual spray presents strong randomness, which is hard for droplets to cover targets uniformly, so using mechanised and intelligent equipment has played a key role in achieving uniform fruit-tree spray in developed regions such as Europe, the United States, Japan and Korea. In Japan and Korea, orchard terrain is mainly hilly and mountainous (Jin et al., 2017). Plant-protection machines mostly utilise miniaturised design with levelling and anti-tipping mechanisms and other safety devices to improve the application efficiency and adaptability to the terrain. However, there are still problems like the imperviousness of dense canopies and the non-uniform distribution of droplets in canopies. In Europe and the United States, the topography of orchards is significantly different from China and Japan. Farm and large-scale planting patterns were generally adopted with deep integration of agronomy and agricultural machinery (Grella et al., 2020), providing the possibility of large plant-protection machinery operations. Among them, air-assisted sprayers are the most widely used devices (Miranda-Fuentes et al., 2017). Although large plant-protection machines show the convenience for plant protection in orchards and have significant application effects compared to manual spray (Liu et al., 2012), they present noticeable problems (Salcedo et al., 2017), such as pesticide overuse, fruit pesticide residues, soil pesticide residues and water pollution (Kira et al., 2018; Kasner et al., 2020). Since the end of the 20th century, many corresponding environmental policies have been promulgated in Europe and the United States, and the use of pesticides has become more stringent. For example, safety quarantine zones must be set up for spray, and pesticides are severely restricted. Therefore, it has been more challenging to develop spray technology to reduce drift and improve the uniformity of droplet distribution. In the 21st century, the target-directional air delivery method gradually replaced the diffuse air delivery method that causes serious drift. The target-directional implement shows a noticeable effect on fruit trees with narrow canopy and uniform height (Song et al., 2012; Niu et al., 2019). However, it is limited by its large size and is only adapted to small canopies and wide row-spacing orchards.

With the advancement of technology, variable spray techniques based on the characteristics of fruit tree canopies have been rapidly developed (He et al., 2011; Cai et al., 2017; Manandhar et al., 2020), and sensors such as LiDAR (Fessler et al., 2020) and depth cameras (Xiao et al., 2017) have been used to acquire fruit tree canopy features (Rosell and Sanz, 2012; Yandún Narváez et al., 2016). Applying pesticides on demand can effectively reduce chemical waste (Miranda-Fuentes et al., 2016). However, there are serious problems. Firstly, in terms of orchard adaptability, after acquiring the characteristics of fruit trees based on sensors, the spray mechanism needs to reach a certain position to deliver droplets onto target locations, which further increases the overall size of the sprayers. (Chen et al., 2011; Liu et al., 2013; Liu et al., 2016) so that they become less adaptable to the orchard environment. In addition, for most large-canopy orchards, canopy closure between rows can directly affect the accuracy of feature sense, even leading to no acquisition of expected canopy features. Inspired by the successful application of plant-protection Unmanned Aerial Vehicles (UAVs) in fields (Zhang et al., 2016), there have been many studies related to plant-protection UAVs in orchard conditions (Wang et al., 2017; Liu et al., 2020; Meng et al., 2020). UAVs can avoid the terrain restrictions that ground sprayers have to suffer but present the disadvantages of the spray for large-canopy fruit trees. The distribution of droplets at the top and bottom of canopies varies highly. With serious row closure, the droplet deposition in the lower part of canopies does not even reach the spraying standard (25 deposits/cm^2^), which still cannot meet the demand for uniform spray in canopies.

**Figure 1:**
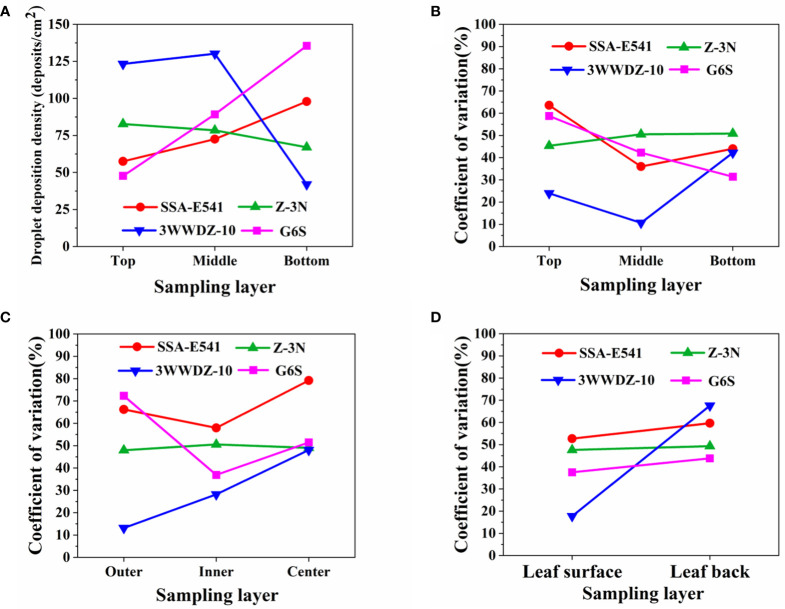
Typical equipment canopy droplet deposition experimental results. **(A)** Vertical longitudinal sampling layer droplet deposition density. **(B)** Vertical longitudinal sampling layer droplet distribution uniformity. **(C)** Horizontal radial sampling layer droplet distribution uniformity. **(D)** Uniformity of droplet distribution on leaf surface and leaf back.

Our team conducted preliminary experiments in two apple orchards in Shanxi Province and Beijing, a mango orchard in Guangxi Province and a citrus orchard in Chongqing Province from June 2018 to April 2021. Typical sprayers for orchards were selected for the experiment, including a ring-shape air-assisted sprayer (model SSA-E541, Wuxi Yifeng Wanshan Technology Co., Ltd.), a tower-shaped air-assisted sprayer (model G6S, Shandong Guohaha Agricultural Machinery Co., Ltd.), a single-rotor plant-protection UAV (model Z-3N, Nanjing Institute of Simulation Technology, Jiangsu Province) and a six-rotor plant-protection UAV (model 3WWDZ-10, Beijing Viga UAV Technology Co., Ltd.). The results in **Figure 1** show that droplets were not uniformly distributed at the top, bottom, inside and outside canopies during single equipment spraying. In particular, during the six-rotor plant-protection UAV spraying, the droplet deposition density in the top layers of canopies was high and uniform, while that in the bottom was poor. Meanwhile, the droplet distribution of the air-assisted sprayer was non-uniform in the top layers and was better in the bottom layers. (Chen et al., 2020; Jiang et al., 2022). These pre-test results were highly consistent with the issue on the basis of the literature review above.

This study proposes a stereoscopic plant-protection method with a corresponding Stereoscopic Plant-protection System (SPS) to improve the uniformity of canopy spray. Numerical simulations using Computational Fluid Dynamics (CFD) and orchard experiments were conducted to determine the optimal operation parameters for the SPS. Then, the experiments of the SPS were conducted. It is a new idea for orchard plant protection, especially for closure orchards, and provides a technical solution to improve the spray uniformity in the canopy of fruit trees.

## 2 Materials and methods

### 2.1 Development of SPS

#### 2.1.1 SPS scheme

**Figure 2:**
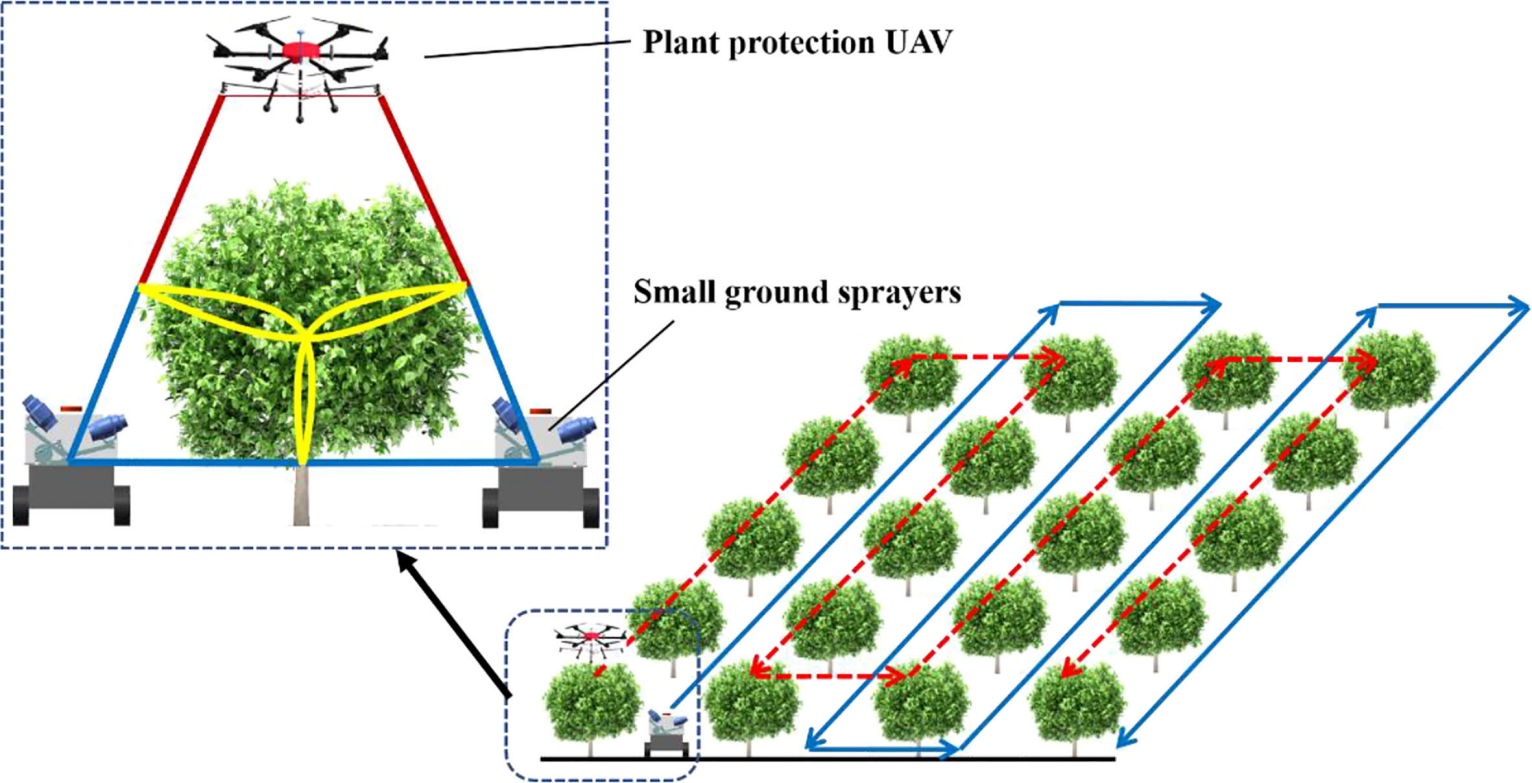
Schematic of stereoscopic plant protection. The dashed line indicates the operation route of the plant-protection UAV, while the solid line is that of the ground sprayer.

As shown in **Figure 1**, the plant-protection UAV and the ground air-assisted sprayer present complementary characteristics for canopy spray. Thus, they were combined to cover the entire tree canopies (**Figure 2**). Plant-protection UAVs were in charge of the upper part of canopies, while small ground sprayers (air-assisted sprayers) focused on the middle and bottom parts.

The plant-protection UAV adopted the canopy top-seeking operation mode (Zhang et al., 2019). It flew directly above the fruit tree canopies, and the spray swath mainly covered the top half of the canopies. The small ground sprayer travelled between the rows of trees, and the spraying range mainly covered the bottom half of the canopies. To prevent the wind fields by the two devices from affecting each other and reducing the spraying effect, the system could select the following three operation modes:

1. sequential independent spraying. One of the devices firstly sprayed. After it was completed, the other one sprayed.
2. simultaneous following spraying. The plant-protection UAV operated first and was followed by the ground sprayer after flying a certain distance (≥spraying width).
3. simultaneous free spraying. The plant-protection UAV and the ground sprayer simultaneously sprayed but did not spray one fruit tree at the same time.

#### 2.1.2 Plant-protection UAV

**Figure 3:**
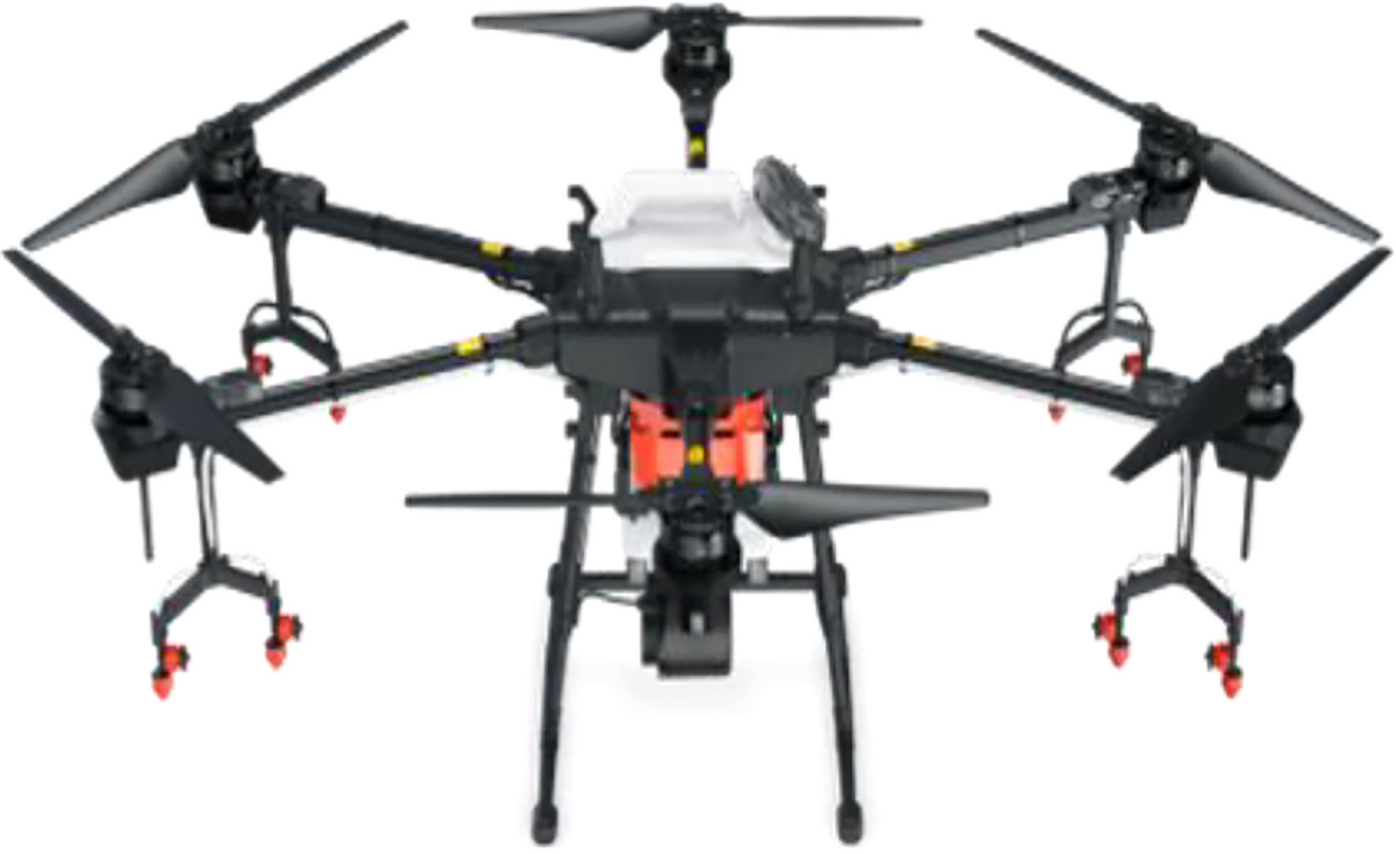
T16 six-rotor plant-protection UAV.

According to the previous study results (Chen et al., 2020), it is known that the droplet deposition density and distribution uniformity of the six-rotor plant-protection UAV on canopies is better than that of the single-rotor one. Therefore, the T16 six-rotor plant-protection UAV (**Figure 3**) produced by Shenzhen DJI Innovation Technology Co., Ltd was exploited for this study. It has a terrain-following function and wide spraying performance, which could ensure a similar spray effect in most cases. Its main parameters are shown in **Table 1**.

**Table 1:** Main parameters of T16 plant-protection UAV.

Categories	Values	Categories	Values
Weight of the whole machine (without battery)	18.50 kg	Medicine tank volume	16.00 L
Nozzle type	SX11001VS	Number of nozzles	8
Operating height (height above the canopy)	1.50∼3.00 m	Maximum spray flow	3.60 L/min
Maximum operating speed	7.00 m/s	Spraying width	4.00∼6.50 m

#### 2.1.3 Small swing-arm sprayer

**Figure 4:**
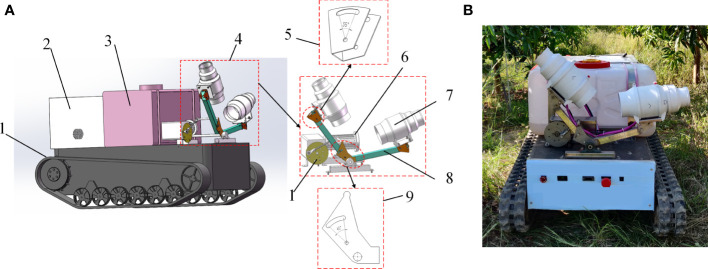
Small swing-arm sprayer. **(A)** Small swing-arm sprayer 3D model. **(B)** Small swing-arm sprayer prototype. 1. Crawler type walking chassis 2. Booster renewal mechanism 3. Liquid tank 4. Swing-arm air-assisted spraying mechanism 5. Angle adjustment parts 6. Direct current reducer motor 7. Piping fan 8. V-shaped swing-arm support bar 9. Driving fan blade.

According to the preliminary investigation of orchard characteristics, a small swing-arm sprayer (**Figure 4A**) was specially developed to spray the lower and middle canopy of fruit trees. The main components consisted of a crawler chassis, a swing-arm air-assisted spraying mechanism, a booster renewal mechanism and a liquid tank. The crawler chassis and the swing-arm air-assisted spraying mechanism were developed earlier, which could autonomously navigate in rows by electrical driving (Liu et al., 2021) and follow spray targets (Jiang et al., 2021), respectively.

The swing-arm air-assisted spraying mechanism was V-shaped and was driven by a DC motor with a drive mechanism to do the swing-arm action. Small pipe fans were installed at the end of each V-shaped swing-arm support bar through the angle adjustment parts. The fans on both sides were controlled independently. Two fan-shaped nozzles were installed at the exit of the fans, respectively. The V-shaped swing-arm support bar was connected by driving fan blades. The angle adjustment parts and the driving fan blade were set with a circular slot, which could adjust the opening and closing angle of the bar. The angle adjustment parts and the driving fan blade were adjustable from 0 to 40° and 0 to 35°, respectively.

In this study, the air-assisted system of the small swing-arm sprayer consisted of small pipe fans. The air volume of the fan was determined according to the displacement principle (Dekeyser et al., 2013). The air-assisted system could effectively reduce the loss of both air volume and energy and enhance the duration of operation.

On the basis of 3D model construction and theoretical parameter calculation, the prototype was developed as shown in **Figure 4B**. Its main technical parameters are shown in **Table 2**.

**Table 2:** The main parameters of the small swing-arm sprayer.

Categories	Values	Categories	Values
Overall dimensions (length × width × height)	2.05 m × 1.10 m × 1.00 m	Maximum spraying width	≤5.50 m
Overall machine mass	500 kg (empty)	Maximum operating speed	0.70 m/s
Power	48V lead battery pack (45Ah)	Maximum fan speed	2500 r/min
Medicine tank volume	150L	Maximum air volume of fan	2304 m^3^/h
Number of nozzles	2	Maximum flow rate of the pump	12 L/min
Nozzle category	Fan spray nozzle	Maximum pressure of the pump	4.50 MPa

### 2.2 Parameter optimisation of SPS based on CFD

In terms of the SPS, the operating parameters of both the UAV and the swing-arm sprayer are essential to improve the spraying performance, whilst the wind fields from these two devices are the key factors affecting the deposition of droplets in canopies (Xu et al., 2017). Thus, CFD was applied to investigate the airflow distribution patterns of these two types of wind fields with fruit trees. The optimal combination of operating parameters with a uniform canopy spraying performance was determined. Based on ANSYS Fluent 18.2, the wind fields of the six-rotor plant-protection UAV and the swing-arm sprayer were numerically simulated.

#### 2.2.1 CFD geometric model construction

**Figure 5:**
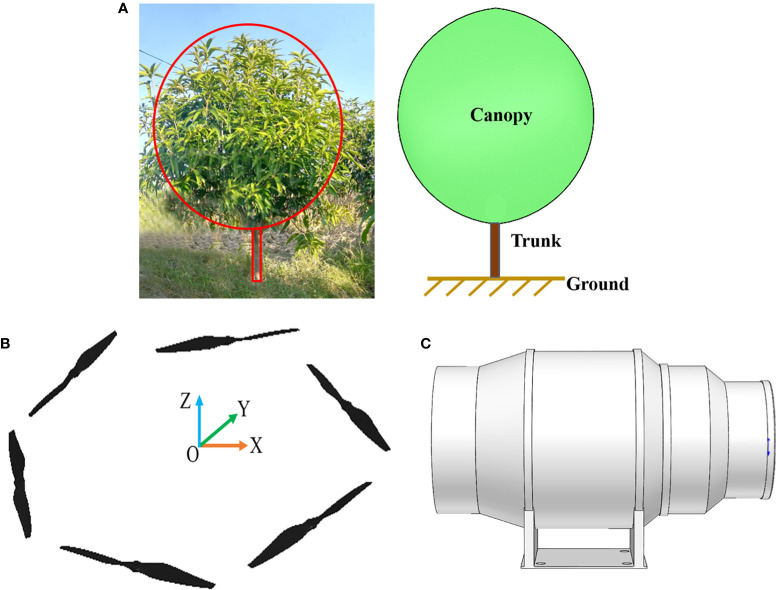
CFD geometric model construction. **(A)** Simplified model of fruit tree. **(B)** Simplified model of plant-protection UAV. **(C)** Simplified model of swing-arm sprayer.

Compared with high computational costs of using entire 3D fruit tree canopy models, using porous medium models to replace fruit tree canopies (Duga et al., 2015; Hong et al., 2018a) has been confirmed by numerous studies for its reliability (Endalew et al., 2009; Salcedo et al., 2015; Duga et al., 2016; Hong et al., 2018b). In this study, fruit tree canopies were represented by a porous medium model, and the hindrance effect of the canopy on airflow was simulated by adding a momentum loss source term in the porous media region. Moreover, the canopy sparseness was characterised by defining different pressure loss coefficients because canopies had various degrees of sparseness and it has different drag magnitudes. The final model is shown in **Figure 5A**, where the full-leaf stage fruit tree was represented by an ellipsoidal canopy and a cylindrical branch trunk. Meanwhile, on the basis of preliminary fruit tree measurements, the canopy pressure loss coefficient, the plant height, the trunk height and the crown width were set as 10.0, 3.5 m, 0.8m and 2.8m, respectively.

In terms of the plant-protection UAV, rotors are the core to generate downwash airflow. Thus, the 3D model of the UAV was appropriately simplified. Only the six rotors were kept, which is acceptable for the simulation (Zhang et al., 2019; Yang et al., 2020). The simplified model of the UAV is shown in **Figure 5B**, where the rotational diameter of the rotor was 609mm.

In terms of the swing-arm sprayer, a similar simplification was conducted. Only the fans were used for simulation, and other components were not considered. The simplified model of the swing-arm sprayer is shown in **Figure 5C**, where the right fan was taken as an example, and the diameter of the wind outlet was 247mm.

#### 2.2.2 Setting of computational areas and boundary conditions

**Figure 6:**
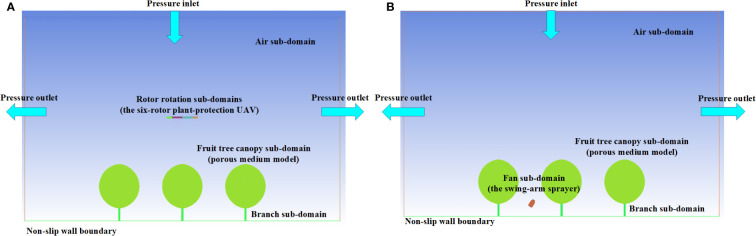
The model calculation area. **(A)** Plant-protection UAV model calculation area. **(B)** Swing-arm sprayer model calculation area.

It is required that numerical simulation conditions should be similar to the actual spraying ones so that simulation results are reliable. In this study, a virtual orchard model was constructed based on the parameters of orchard investigations, the model calculation area was 20.0 m × 15.0 m (long ×wide) with a height of 13.0 m (**Figure 6**), so airflow could be fully developed. The model included the fruit tree canopy subdomain and a branch subdomain. The fruit tree branch subdomain did not need to be solved, so it was removed during preprocessing and set as the wall boundary. Only the outlet boundary was kept.

The air sub-domain and rotor rotation sub-domains were included in the simulation of the six-rotor plant-protection UAV. The ‘interface’ boundary condition was applied for the interface between the air sub-domain and the rotor rotation sub-domains. The rotor rotation (rotational speed 2500 r/min) sub-domain was processed by slip grids. Meanwhile, the upper boundary of the air sub-domain was the pressure inlet, the lower boundary of the ground was set to the non-slip wall boundary, and the other boundaries were the pressure outlets. The rotor was 2.0m away from the top of the fruit tree canopies.

The air sub-domain and fan sub-domains were included in the simulation of the swing-arm sprayer. The fan was located at the central line of the row spacing, about 2.0 m from the tree trunk and 0.6 m above the ground. For the setting of the solution parameters, only the external flow field of the fans was concerned. Moreover, the no-slip wall boundary was used for the air subdomain, and the rest of the boundaries were set as pressure outlets.

Meshing was conducted after the geometric model and the computational areas were determined. Non-structural tetrahedral meshes applicable to complex entities were used for gridding. The mesh numbers for the plant-protection UAV and swing-arm sprayer simulation were 8112602 and 4694187, respectively. No negative meshes and left-hand meshes existed, so the meshes were used for calculation.

In terms of solution, the renormalization group (RNG) *κ*-*ε* turbulence model was selected, and the control equations were discretised by the finite volume method. The pressure-velocity coupling was chosen from the Pressure-Implicit with Splitting of Operators (PISO) algorithm. The pressure interpolation format was chosen from the PRESTO! format for high-speed rotating and porous media. The second-order windward pair momentum, turbulent kinetic energy and turbulent dissipation rate were discretised in the spatial domain.

### 2.3 Orchard experiments of SPS

#### 2.3.1 Experimental site and sprayers

**Figure 7:**
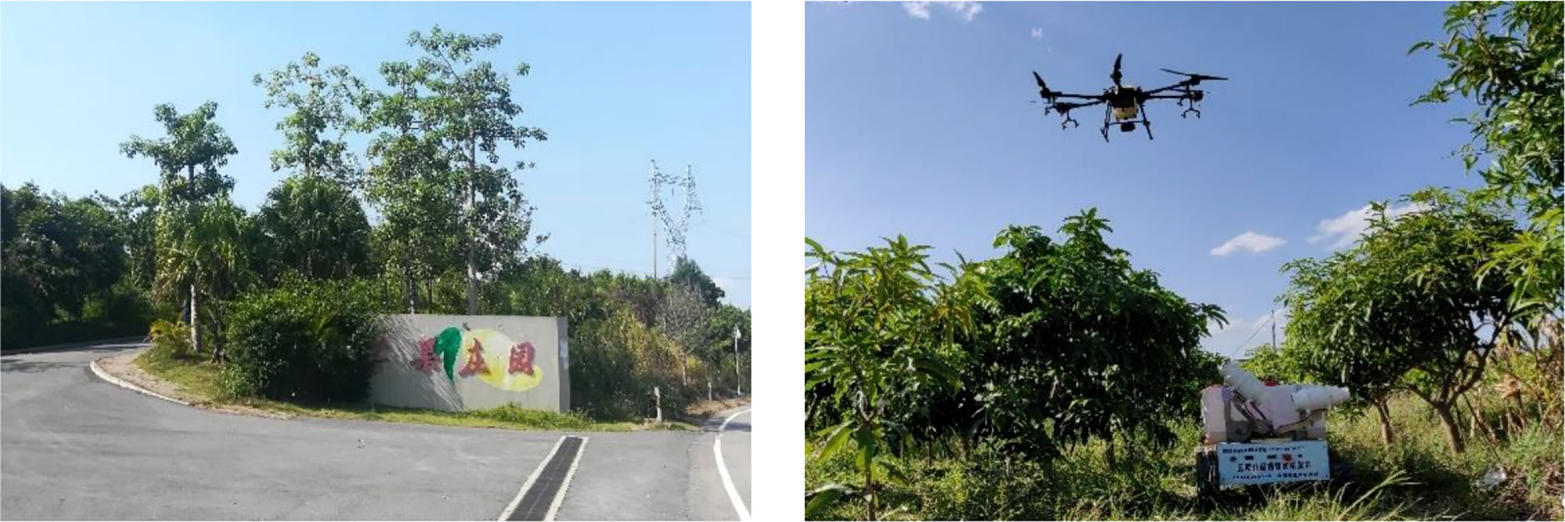
Experimental scenarios.

The experiment was conducted in August 2020 in mango orchards in Tianyang District, Baise City, Guangxi Zhuang Autonomous Region (**Figure 7**). The environmental temperature during the experiment was about from 28°C to 32°C, and the humidity was about from 45% to 49%. The orchards were planted in the conventional mode. The row spacing was 4.5 m, the plant interval was 3.5 m, and the tree height was about 4.5 m. The trees were about 30 years old, and the canopies were large and closed in some areas. The UAV (**Figure 3**) and the sprayer (**Figure 5**) were employed in the experiments.

In addition, the wind speed and volume meter, AR856 produced by Shenzhen Franken Electronics Co., Ltd., and temperature and humidity meter produced by Deloitte Group Co., Ltd. were used to monitor and record meteorological parameters such as wind speed, wind direction, temperature and humidity.

#### 2.3.2 Experimental scheme

The experiments were conducted according to the standards NY/T 992-2006, ‘The operation quality for air-assisted orchard sprayer’, and JB/T 9782-2014, ‘Equipment for crop protection - General test methods’.

1) Sampling point arrangement

**Figure 8:**
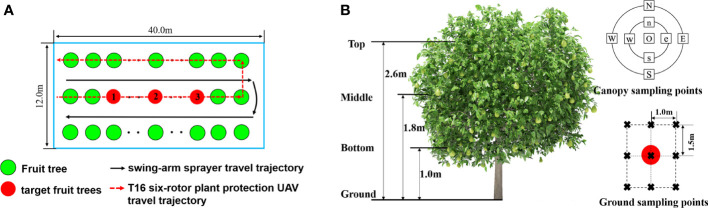
Sampling scheme. **(A)** Selection of target fruit trees. **(B)** Sampling point layout scheme.

The experimental scheme is shown in **Figure 8**. Three fruit trees with similar shape, height and canopy size were selected as target fruit trees in the experimental area (**Figure 8A**). The target fruit trees were far from the start and end of rows to reduce the errors caused by the devices slowing down and turning.

The scheme of canopy droplet sampling points is shown in **Figure 8B**. In the canopy, according to the height and shape of each target tree, vertical sampling was divided into top, middle and bottom layers, and horizontal radial sampling was divided into the centre, inner and outer layers relative to the location of the trunk. One sampling point was placed in the centre layer of canopies and marked as O. Four sampling points were placed in the inner and outer layers of canopies, marked as e, s, w, n and E, S, W, N, respectively. Thus, there were 27 sampling points in canopies. On the ground, 9 sampling points were set with the trunk of the tree as the centre.

Water-sensitive papers (76 mm × 26 mm) were exploited to collect droplets, laid on both sides of leaves on each sampling point, so 54 pieces were used in total. Each ground sampling point arranged a water-sensitive paper and made the water-sensitive paper face up. The spray solution was water without solids in suspension at the normal temperature.

2) Optimization of operational parameters based on orthogonal experiment

The previous CFD simulation had determined both the proper operation height of the six-rotor plant-protection UAV and the appropriate swing-arm angle of the swing-arm sprayer. Hence, a three-factor with three-level orthogonal experiment was conducted to find the optimal operation speed. The factor level of the orthogonal experiment is shown in **Table 3**.

**Table 3:** Three-factor with three-level orthogonal experiment table for the SPS.

Experimental group	Factor A: Swing-arm sprayer operating speed (m/s)	Factor B: T16 plant-protection UAV operating height (m)	Factor C: T16 plant-protection UAV operating speed (m/s)
1	0.40 (1)	2.00 (1)	1.00 (1)
2	0.40 (1)	2.50 (2)	1.50 (2)
3	0.40 (1)	3.00 (3)	2.00 (3)
4	0.50 (2)	2.00 (1)	1.50 (2)
5	0.50 (2)	2.50 (2)	2.00 (3)
6	0.50 (2)	3.00 (3)	1.00 (1)
7	0.60 (3)	2.00 (1)	2.00 (3)
8	0.60 (3)	2.50 (2)	1.00 (1)
9	0.60 (3)	3.00 (3)	1.50 (2)

A1 refers to the first level of factor A, that is, A1 is 0.40m/s; B1 refers to the first level of factor B, that is, B1 is 2.00 m; other factor levels are expressed in the same way, e.g., B2, C3, etc.

The sequential independent mode was used for the experiment. After setting water-sensitive paper, the swing-arm sprayer was firstly enrolled in the test. When the sprayer completed the test, the T16 UAV started.

3) Effect verification of the SPS based on trials

Based on the CFD simulation and the orthogonal experiment, the optimal operating parameters of the SPS were determined. They were selected for the effect verification of the SPS, comparing the spraying effect of the SPS with that of the T16 plant-protection UAV and the swing-arm sprayer. The operation parameters for the single-device experiment were the same as that for two-device one.

#### 2.3.3 Data analysis

All the water-sensitive paper was processed by the following steps:.

♦ All the water-sensitive paper was scanned with LASERJET PRO MFP M132 to obtain the corresponding scan images. Then, the images were read by DepositScan ™ droplet analysis software to get indices such as droplet deposition, deposition density and coverage. All the data were recorded in an Excel table.
♦ The coefficient of variation was calculated by using the equations from (1) to (3) to analyse the droplet distribution uniformity. SPSS 21.0 and Origin 9.1 software were used for data processing and graph plotting.

(1) $$\bar{q}=\frac{\Sigma q_{i}}{n}$$

(2) $$S=\sqrt{\frac{\Sigma {\left(q_{i}-\bar{q}\right)}^{2}}{n-1}}$$

(3) $$CV(\%)=\frac{s}{\bar{q}}\times 100$$

where, *q_i_* is the *i*-th sampling point droplet deposition density, deposits/cm^2^; $\bar{q}$ is the average value of sampling point droplet deposition density, deposits/cm^2^; n is the number of sampling points; S is the standard deviation of droplet deposition density, deposits/cm^2^, and *CV* (%) is the coefficient of variation.

## 3 Results and discussion

### 3.1 Results and analysis of CFD simulation

#### 3.1.1 Wind field distribution of the six-rotor plant-protection UAV

(1) Time-dependent characteristics of wind field speed

**Figure 9:**
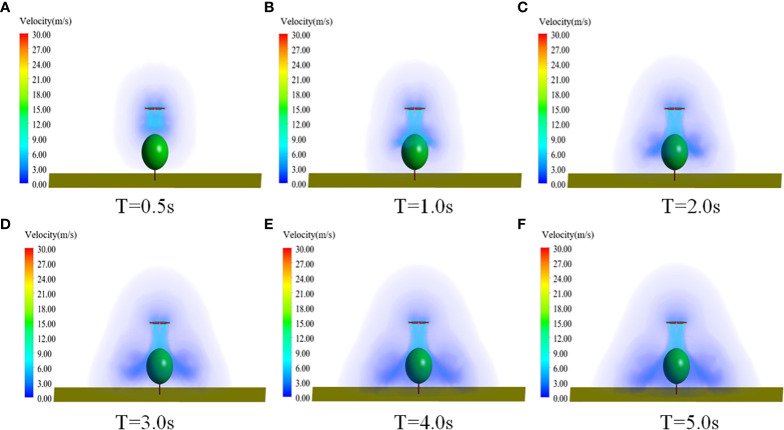
Wind field speed distribution of six-rotor plant-protection UAV at different times. **(A)** T=0.5s. **(B)** T=1.0s. **(C)** T=2.0s. **(D)** T=3.0s. **(E)** T=4.0s. **(F)** T=5.0s.

**Figure 9** shows the speed distribution of the rotor wind field at different moments, respectively. It can be seen that the rotor airflow kept extending downward with increasing time. At 0.5 s, the rotor airflow approximately reached canopies. At 1.0s, the rotor airflow covered the top of canopies. At 5.0 s, the wind field had not yet reached spreading along the ground, although some of it touched the ground. Therefore, a six-rotor plant-protection UAV was used for fruit tree spraying, the height from the top of the canopy was 2.0m. Meanwhile, the plant-protection UAV stayed at least 4.0s after take-off and then started operation.

(2) Wind speed distribution of different locations in canopies

The simulated fruit trees were divided into top, middle and bottom layers at a distance of 2.3m, 3.4m and 4.5m from the center of the plant-protection UAV, and 9 sample points were selected uniformly in each layer (the sample point distribution scheme was the same as the foliar sampling point layout scheme in Section 2.1) to obtain the maximum airflow velocity in the vertical direction (Z direction) at each sample point (**Table 4**).

**Table 4:** Velocity distribution of each layer within the canopy.

Location	Sample point speed (m/s)									Average speed (m/s)
	1	2	3	4	5	6	7	8	9	
Top layer	3.11	5.05	3.88	5.14	2.02	2.23	3.03	2.36	2.18	3.22
Middle layer	0.42	0.41	0.37	0.35	0.61	0.57	0.59	0.57	0.68	0.51
Bottom layer	0.10	0.09	0.09	0.10	0.10	0.10	0.10	0.10	0.11	0.10

As shown in **Table 4**, the average speeds of the top, middle and bottom layers inside canopies were 3.22 m/s, 0.51 m/s and 0.10 m/s, respectively, with a decreasing trend from top to bottom. The average wind speed in the bottom layers was minimal, which could hardly carry and transport droplets.

#### 3.1.2 Wind field distribution law of the swing-arm sprayer

**Figure 10:**
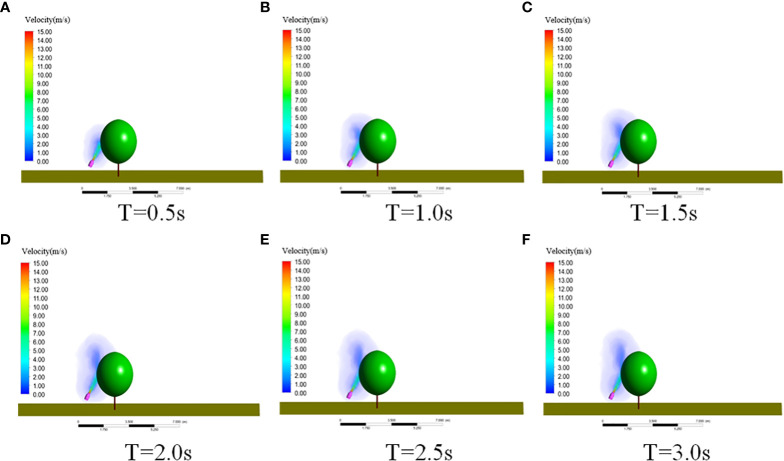
Wind speed distribution of the swing-arm sprayer at different times. **(A)** T=0.5s. **(B)** T=1.0s. **(C)** T=1.5s. **(D)** T=2.0s. **(E)** T=2.5s. **(F)** T=3.0s.

**Figure 10** shows the wind field velocity distribution of the fan at different moments, indicating that canopies had an obvious blocking effect on the fan airflow. It can be seen that at 0.5 s, the fan airflow reached canopies. At 1.0 s, the airflow appeared to roll up around canopies because of the blocking effect. The rolled-up airflow gradually increased and kept stable at about 2.5 s. Therefore, the swing-arm sprayer could start spraying after the fan was turned on for 2.5 s.

#### 3.1.3 Optimal operation parameters of the SPS

**Figure 11:**
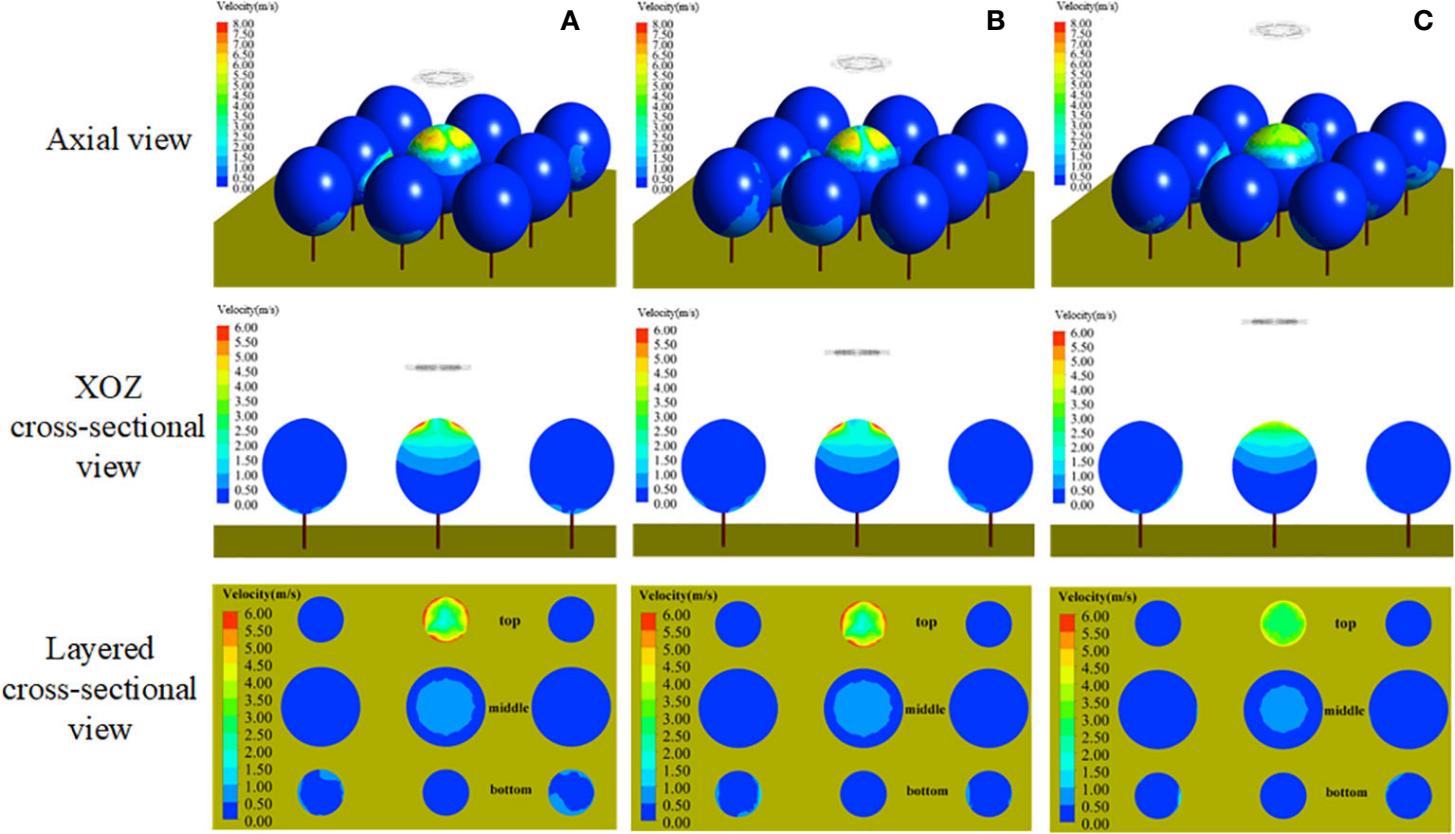
Air speed distribution of plant-protection UAV at different heights. **(A)** Operating height 1.5m. **(B)** Operating height 2.0m. **(C)** Operating height 3.0m.

**Figure 11** shows the velocity distribution of the stable wind field of the UAV rotors at different operating heights (height to the top of the canopy). The rotor airflow reached the canopy surface in a centrosymmetric pattern, and the operating height caused the change of the airflow to the target. The airflow area to targets gradually decreased as the operating height increased. In the range from 1.5 m to 2.0 m, the airflow velocity changes in the canopy were not obvious, and the optimal operation height should be selected in this range.

**Figure 12:**
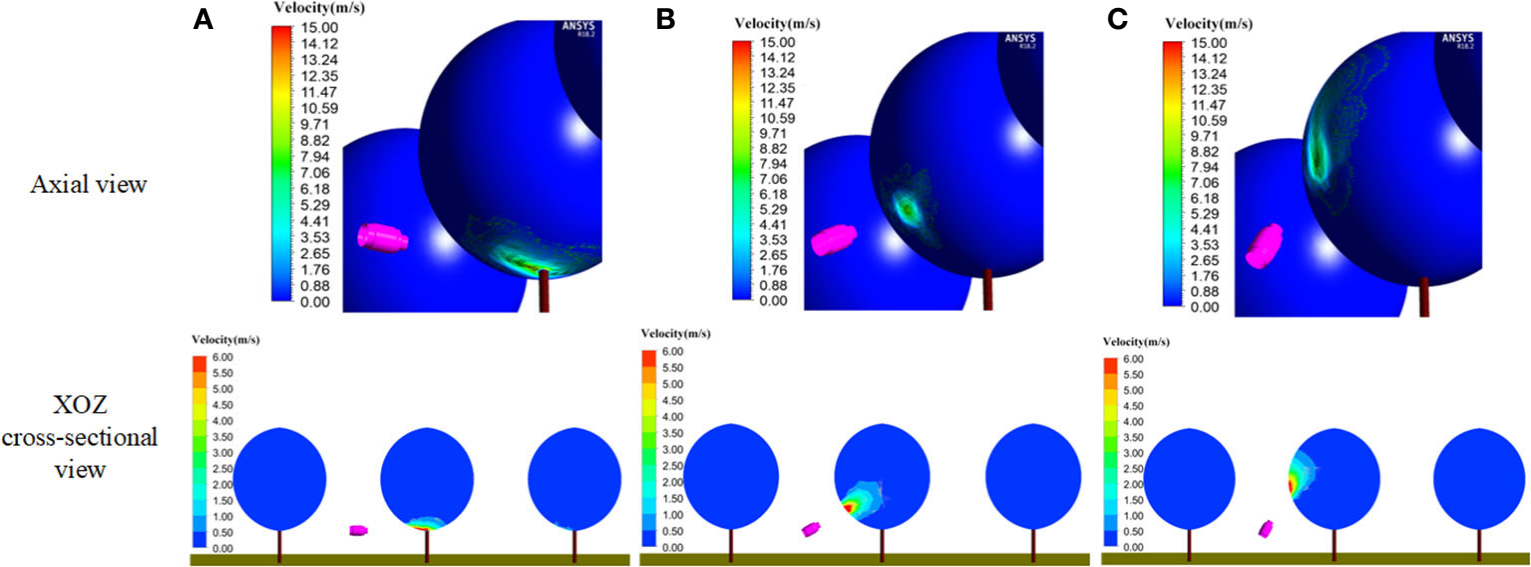
Fan air speed distribution under different swing-arm angles. **(A)** Swing-arm angle 0°. **(B)** Swing-arm angle 30°. **(C)** Swing-arm angle 60°.

**Figure 12** shows the velocity distribution of the stable wind field of the sprayer fan at different swing-arm angles. During spraying, the area covered by the airflow from the fan to the target gradually increased gradually with the swing-arm angle started from 0°. When the swing-arm angle was certain, the airflow velocity inside canopies gradually decreased as the canopy depth increased. It basically covered the lower half side (left side) of canopies, and the airflow mostly spread uniformly in the range from 1.5 m/s to 3.5 m/s, which is beneficial to the uniform distribution of droplets.

**Figure 13:**
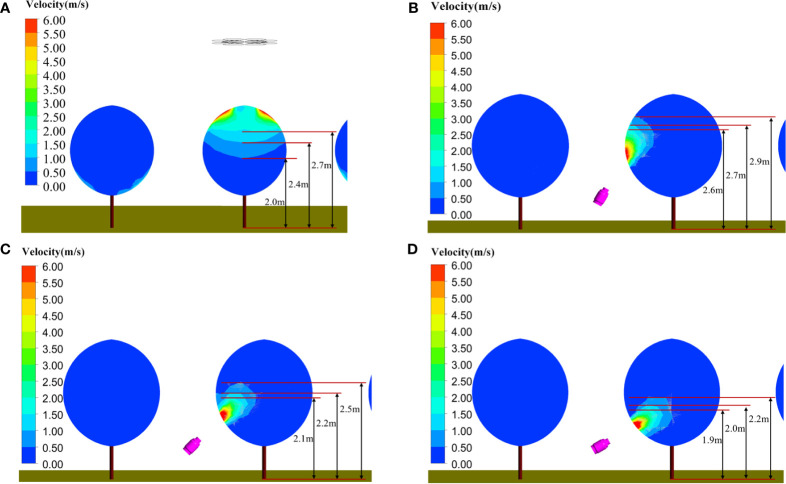
Comparison of canopy wind field coupling under different operating parameters. **(A)** Plant-protection UAV operation height 2.0 m. **(B)** Swing-arm angle of swing-arm sprayer 60°. **(C)** Swing-arm angle of swing-arm sprayer 45°. **(D)** Swing-arm angle of swing-arm sprayer 30°.

Based on the above simulation results, the comparative effect of the wind field coupling in the stereoscopically applying canopy with different operating parameters is given in **Figure 13**. When the operation height of the UAV was 2.0 m, the rotor airflow speed was between 0.50 m/s and 1.00 m/s in the range of canopy height from 2.0 m to 2.4 m, the rotor airflow speed was between 1.00 m/s and 1.50 m/s in the range of canopy height from 2.4 m to 2.7 m, the rotor airflow speed was above 1.5 m/s in the range of canopy height from 2.7 m, the rotor airflow speed was above 1.5 m/s in the range of canopy height from 2.7 m. When the swing-arm angle of the swing-arm sprayer was 60°, the fan airflow speed of canopy height below 2.6 m was above 1.5 m/s, the fan airflow speed between 2.6 m and 2.7 m was from 1.00 m/s to 1.50 m/s, and the fan airflow speed of canopy height above 2.7m rapidly became smaller.

Therefore, when the maximum swing-arm angle of the swing-arm sprayer was 60° and the operation height of the plant-protection UAV was 2.0 m, the wind fields of the two devices could be coupled enough.

### 3.2 Results and analysis of the orthogonal experiment

The orthogonal test results are shown in from **Table 5** to **Table 7**.

**Table 5:** Results of vertical longitudinal droplet deposition distribution in the canopy.

Experimental group	Droplet deposition density (deposits/cm^2^)			Coefficient of variation/%		
	Top layer	Middle layer	Bottom layer	Top layer	Middle layer	Bottom layer
1	90.10	99.60	106.80	43.75	16.08	10.54
2	73.80	91.90	95.40	44.82	31.39	32.79
3	65.60	103.90	95.20	82.25	31.90	45.13
4	49.50	61.30	79.60	87.00	95.00	39.00
5	63.20	53.30	70.30	53.58	17.94	33.16
6	52.90	80.60	88.50	61.75	34.28	28.25
7	66.80	91.10	94.20	42.03	33.23	39.85
8	58.90	101.10	65.90	39.84	25.60	25.91
9	78.00	56.20	52.90	61.22	76.59	64.88

**Table 6:** Canopy vertical longitudinal droplet deposition density range analysis.

Indicators	Factor A			Factor B			Factor C		
	Top layer	Middle layer	Bottom layer	Top layer	Middle layer	Bottom layer	Top layer	Middle layer	Bottom layer
*K*_1_	229.50	295.40	297.40	206.40	252.00	280.60	201.90	281.30	261.20
*K*_2_	165.60	195.20	238.40	195.90	246.30	231.60	201.30	209.40	227.90
*K*_3_	203.70	248.40	213.00	196.50	240.70	236.60	195.60	248.30	259.70
$\bar{K_{1}}$	76.50	98.50	99.10	68.80	84.00	93.50	67.30	93.80	87.10
$\bar{K_{2}}$	55.20	65.10	79.50	65.30	82.10	77.20	67.10	69.80	76.00
$\bar{K_{3}}$	67.90	82.80	71.00	65.50	80.20	78.90	65.20	82.80	86.60
Range	21.30	33.40	28.10	3.50	3.80	16.30	2.10	24.00	11.10

K_i_ indicates the sum of the experimental results corresponding to each factor at level i, $\bar{K_{i}}$ indicates the mean of the experimental results corresponding to each factor at level i.

**Table 7:** Canopy vertical longitudinal fog droplet distribution uniformity range analysis.

Indicators	Factor A			Factor B			Factor C		
	Top layer	Middle layer	Bottom layer	Top layer	Middle layer	Bottom layer	Top layer	Middle layer	Bottom layer
*K*_1_	170.82	79.37	88.46	172.78	144.31	89.39	145.34	75.96	64.70
*K*_2_	202.33	147.22	100.41	138.24	74.93	91.86	193.04	202.98	136.67
*K*_3_	143.09	135.42	130.64	205.22	142.77	138.26	177.86	83.07	118.14
$\bar{K_{1}}$	56.94	26.46	29.49	57.59	48.10	29.80	48.45	25.32	21.57
$\bar{K_{2}}$	67.44	49.07	33.47	46.08	24.98	30.62	64.35	67.66	45.56
$\bar{K_{3}}$	47.70	45.14	43.55	68.41	47.59	46.09	59.29	27.69	39.38
Range	19.74	22.61	14.06	22.33	23.12	16.29	15.90	42.34	23.99

K_i_ indicates the sum of the experimental results corresponding to each factor at level i, $\bar{K_{i}}$ indicates the mean of the experimental results corresponding to each factor at level i.

In terms of droplet deposition density, deposition uniformity and range analysis (**Tables 5**–**7**), it is known that the results of experiment group 1 (A1B1C1 group) were better than the others, demonstrating an optimal spraying performance.

According to **Table 6**, the factors affecting the droplet deposition density in order of priority were the speed of the swing-arm sprayer, the operating height and the operating speed of the T16 UAV.

According to **Table 7**, for the top of canopies, the order of factors affecting the uniformity of droplet distribution was T16 plant-protection UAV operation height, swing-arm sprayer speed and T16 plant-protection UAV operation speed. For the middle and lower part of canopies, that was UAV operation speed, UAV operation height and swing-arm sprayer travel speed.

According to the analysis of the above experimental results, the optimal operation parameters of SPS were: a speed of 0.4 m/s and 1.0 m/s for the swing-arm sprayer and the T16 plant-protection UAV, respectively, and an operating height of 2.0 m for the UAV.

### 3.3 Results and analysis of the verification trials

**Figure 14:**
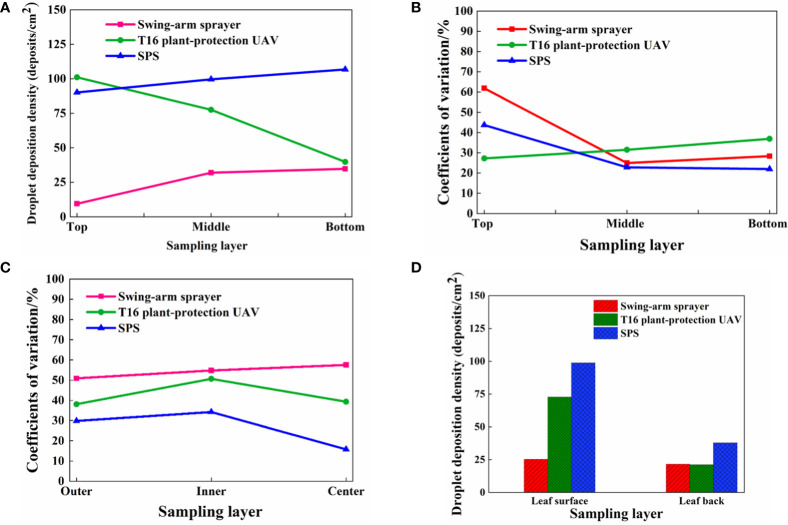
Results of the validation experiment of the SPS. **(A)** Vertical longitudinal sampling layer droplet deposition density. **(B)** Vertical longitudinal sampling layer droplet distribution uniformity. **(C)** Horizontal radial sampling layer droplet distribution uniformity. **(D)** Density of droplet deposition on leaf surface and leaf back. The values in the figure are the average values of the sampling points when not stated. For example, the density of droplet deposition in the top of canopies was the means of the values of all the corresponding positions.

The results of the verification trials are shown in **Figure 14**.

The SPS could significantly increase droplet deposition density. When the T16 plant-protection UAV operated independently, the canopy droplet deposition density decreased from top to bottom. The maximum droplet deposition density was 101 deposits/cm^2^ at the top layers, and its range was nearly 61 deposits/cm^2^. When the swing-arm sprayer operated independently, it was less than 10 deposits/cm^2^, and the density in the middle and lower layers was closer and reached the spray quality requirements. The maximum density range was 24 deposits/cm^2^. When the SPS operated, the density range was from 90 to 107 deposits/cm^2^, and the maximum density range was only 17 deposits/cm^2^.

The uniformity of droplet distribution of the SPS was generally better than that of the T16 UAV and the swing-arm sprayer. It was only weaker than the T16 UAV in the upper canopy layer. The coefficient of variation was 16.1% and 10.5% in the middle and lower canopy layers, 38.3% higher than that of the conventional air-assisted sprayer in the corresponding positions. The horizontal radial droplet distribution of the SPS was better than that of both the T16 UAV and the swing-arm sprayer. The variation coefficients of each canopy layer of the SPS from the outside to the inside were 29.8% 34.2% and 15.8%.

The SPS performed better than the T16 plant-protection UAV and swing-arm sprayer in terms of droplet deposition density on the front and back of the leaves. The droplet density on both sides was higher than the theoretical application requirement of 25 deposits/cm^2^.

**Figure 15:**
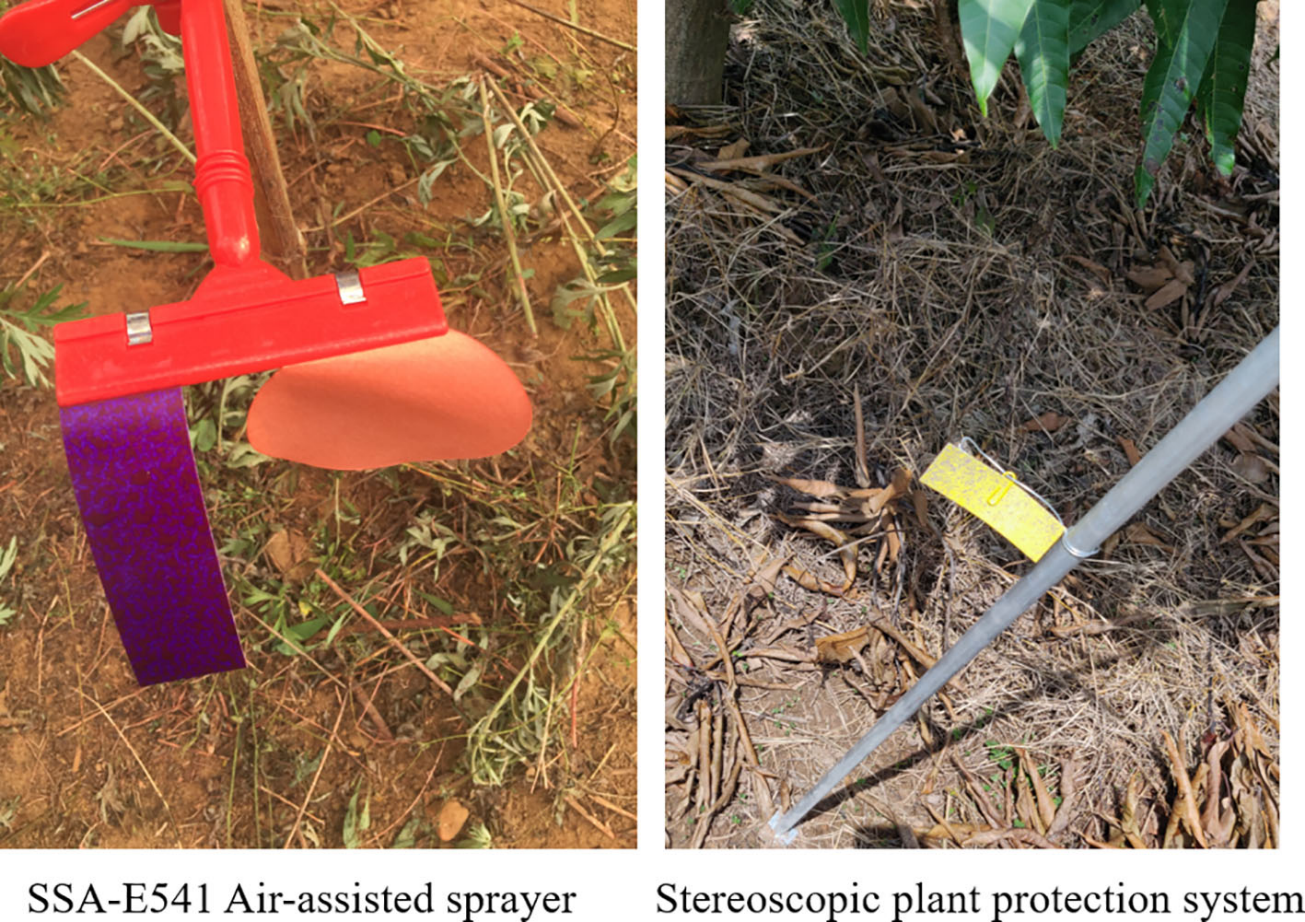
Comparison of ground loss.

The ground loss of the SPS and the SSA-E541 air-assisted sprayer were compared. As shown in **Figure 15**, the ground loss of the SPS reduced significantly.

According to the analysis above, it is indicated that the spraying performance of the SPS was obviously improved. The SPS could be a new way for mechanisation of orchard plant protection, especially for the orchards in hilly areas.

### 3.4 Discussions

The trafficability of the miniaturised sprayers and the high flexibility of plant-protection UAVs could effectively solve the difficulties of a) large machines entering and leaving and b) the non-uniform application of small machines.

Compared with field crops, fruit trees have the characteristics of large canopies. The phenomenon of depression between rows is common, especially in mature fruit trees and traditional orchards. There is the problem of impermeable and uneven canopies during spraying, and it is difficult for sprayers to be applied in such orchards. To solve the above issues, this study proposes a layered spraying method, using a plant-protection UAV and a small ground sprayer to spray different locations of canopies, respectively. This method ensures good passability in orchards and improves spray uniformity in canopies. The feasibility of this method was further verified through actual orchard trials.

However, there are still some shortcomings:

1. this study obtained the optimal parameters of SPS by using CFD and orthogonal tests. Nonetheless, the parameter selection took a lot of time, and the subsequent experiments can be performed to further optimization of the best parameter determination method and improve the efficiency.
2. In this study, the experiments were conducted in mango orchards (big canopy). The reliability of application parameters needs to be further verified for orchards with vertical planting patterns (high canopies).
3. precision application methods (such as target application and variable application) can be combined to improve pesticide use further and reduce waste and environmental pollution.

## 4 Conclusion

To solve the problems that the spray droplet distribution of plant-protection UAV on the canopy is ‘more on top and less on the bottom’, while the ground sprayer is ‘less on top and more on the bottom’, an asynchronous stratified stereoscopic plant-protection method combining small ground sprayer and a plant-protection UAV is proposed. The main conclusions are as follows:

1. The overall scheme of stereoscopic plant-protection was defined based on the spraying requirements. The plant-protection UAV was selected and a small swing-arm sprayer was designed. The SPS consisting of a T16 six-rotor plant-protection UAV and a small swing-arm sprayer was developed.
2. The CFD-based optimisation of the operational parameters of the SPS was conducted. The wind field distribution characteristics of the plant-protection UAV and the swing-arm sprayer were clarified, and the coupling effects of the canopy wind field of stereoscopic spraying were analyzed. The theoretical operating parameters of the SPS for uniform application to the canopy of fruit trees were identified. The operating height of the plant-protection UAV was 2.0 m, and the swing-arm angle of the swing-arm sprayer was 60°.
3. Based on CFD numerical simulation, a three-factor with three-level orthogonal experiment was conducted to identify the optimal parameters of the SPS. The speed of the swing-arm sprayer was 0.4 m/s, the operating height of the T16 plant-protection UAV was 2.0 m, and the operating speed was 1.0 m/s, respectively. They were selected for the verification experiments of the SPS. The results showed that the SPS had a vertical longitudinal droplet deposition density of 90-107 deposits/cm^2^ in canopies, and the coefficients of variation of uniformity in the top, middle and lower layers were 43.7%, 16.1% and 10.5%, respectively, and the uniformity was 38.3% higher than conventional air-assisted sprayers. The coefficient of uniformity variation of the horizontal radial canopy from outer to central layers was 29.8%, 34.2% and 15.8%, respectively. The uniformity of application of the SPS in the upper, lower, inner and outer canopies of fruit trees were significantly improved, while the density of droplets deposited on both sides of the leaves was more than 25 deposits/cm^2^, and could meet the spray requirements.

The SPS proposed in this paper can provide an adequate technical means and solution for uniform application to large canopy fruit trees. Meanwhile, the high mobility of plant-protection UAVs and the high trafficability of small swing-arm sprayers between orchard rows can solve the problem of the difficulty of entering and leaving the orchard when using large plant-protection equipment.

## Data availability statement

The raw data supporting the conclusions of this article will be made available by the authors, without undue reservation.

## Author contributions

SJ: Data curation, tests, and writing- original draft. BC: Formal analysis, data curation, visualization, tests. WL: Tests and validation. SY: Methodology, writing - review & editing. YZ: Supervision, methodology and resources. XL: Methodology and writing - review & editing. All authors contributed to the article and approved the submitted version.

## Funding

This study is supported and funded by the Yantai Locality and University Cooperation Development Project (2021XDRHXMPT29), the National Key R&D Program of China (2018YFD0700603), and the National Natural Science Foundation of China (NSFC) (32171901).

## Acknowledgments

Appreciation to Guangxi Agricultural Machinery Research Institute Co., Ltd. for their help in the experiment process.

## Conflict of interest

The authors declare that the research was conducted in the absence of any commercial or financial relationships that could be construed as a potential conflict of interest.

## Publisher’s note

All claims expressed in this article are solely those of the authors and do not necessarily represent those of their affiliated organizations, or those of the publisher, the editors and the reviewers. Any product that may be evaluated in this article, or claim that may be made by its manufacturer, is not guaranteed or endorsed by the publisher.
